# Ventilatory defects and treatable traits in very elderly patients

**DOI:** 10.1177/00368504211013171

**Published:** 2021-04-30

**Authors:** João Gaspar-Marques, Teresa Palmeiro, Iolanda Caires, Paula Leiria Pinto, Nuno Neuparth, Pedro Carreiro-Martins

**Affiliations:** 1 NOVA Medical School/Comprehensive Health Research Centre (CHRC), Lisbon, Portugal; 2 Immunoallergology Service, Dona Estefânia Hospital, Central Lisbon Hospital Centre, EPE, Lisbon, Portugal

**Keywords:** Aged, comorbidity, lung diseases, obstructive, respiratory disorders

## Abstract

Though the approach used to classify chronic respiratory diseases is changing to a treatable-traits (TT) approach, data regarding very elderly patients is lacking. The objectives of this study were to assess TT frequency in very elderly patients and to study the link between extrapulmonary TT and ventilatory defects. Individuals (≥75 years) residing in elderly care centres answered a standardised questionnaire, underwent spirometry, atopy and fractional exhaled nitric oxide assessments and had their blood pressure and peripheral pulse oximetry measured. Pulmonary, extrapulmonary and behavioural TT were evaluated. Outcome variables were an airflow limitation (post-bronchodilator z-score FEV_1_/FVC<−1.64) and a restrictive spirometry pattern (z-score FEV_1_/FVC ≥ +1.64 and z-score FVC<−1.64). Seventy-two percent of the individuals who took part in the study (*n* = 234) were women, and the median age of participants was 86 (IQR: 7.4). At least one pulmonary TT was identified in 105 (44.9%) individuals. The most frequent extrapulmonary TTs were: persistent systemic inflammation (47.0%), anaemia (34.4%), depression (32.5%) and obesity (27.4). Airflow limitation was exclusively associated with smoking (OR 5.03; 95% CI 1.56–16.22). A restrictive spirometry pattern was associated with cognitive impairment (OR: 3.89; 95% CI: 1.55–9.79). A high frequency of various TTs was found. The novel association between a restrictive spirometry pattern and cognitive impairment highlights the urgency of clinical research on this vulnerable age group.

## Introduction

Population ageing is the triumph of advances made in public health, medicine and economic and social development over diseases that have limited human lifespans throughout history.^1^ According to the United Nations’ publication ‘World Population Prospects of 2019’, by 2050, one in six people (16%) in the world will be over the age of 65, up from one in 11 in 2019 (9%).^1^ In some regions, like Europe and North America, by 2050, one in four people are predicted to be over the age of 65.^1^ Furthermore, the number of people aged 80 or over is projected to triple, from 143 million in 2019, to 426 million in 2050.^1^ Unfortunately, despite the growing percentage of older people in the population, they remain underrepresented in clinical research.^2^ This is particularly true for those living with multimorbidity, frailty or dementia and those who live in care homes.^2^ With the growing phenomenon of population ageing, the need for robust, high-quality health research that includes older people has never been greater,^2^ as the extrapolation of data and treatment recommendations from studied to unstudied populations can result in catastrophic consequences and increased costs.^3^

Chronic respiratory diseases (CRDs) are common, and – relatively – increasing, causes of disability and death.^4^ The physiologic, cellular and immunologic changes that occur due to ageing contribute to the development of lung disease.^5^ Although the prevalence of CRDs has been increasing as the population ages, it may still be underestimated in older persons.^5^

The concept of disease classification in CRDs has changed considerably in recent years.^6,7^ Standard classifications based on symptoms, signs and functional abnormalities are being substituted by the treatable trait approach,^6,7^ a label-free, precision medicine approach to the diagnosis and management of CRDs.^6^ This approach is based on identifying treatable traits in each patient, which might be treatable based on ‘phenotypic’ recognition or a deep understanding of the critical causal pathways.^6^ Treatable traits are divided into the following categories: pulmonary, extrapulmonary and behavioural/lifestyle risk factors.^6^ From a patient perspective, it is important to recognise that each patient may have more than one treatable trait, and that, in fact, many do. One of the potential strengths of this approach is that it does not rest on the assumption that the diagnosis (e.g. asthma or chronic obstructive pulmonary disease) is well-established and clear, circumstances that not often the case in clinical practice. In elderly patients, the issue might be particularly pertinent as typical symptoms of CRDs can often also have non-pulmonary origins.^8^ The treatable traits approach relies on specific diagnostic criteria being defined for these ‘traits’, which may have a considerable impact on patient treatment should the more favourable therapeutic response expected be evidenced.^6^ Information about the interrelations between treatable traits is lacking. This fact is particularly evident in very elderly patients and ventilatory defects.

The aim of this study was to investigate the frequency of treatable traits in a general population of very elderly individuals and to find the link between extrapulmonary and behavioural/lifestyle risk factors and ventilatory defects.

## Methods

### Study design, setting and participants

The OLDER (Obstructive Lung Diseases in the Elderly) study took place in Lisbon, Portugal. It was an observational study, divided into three phases. This study shall report results from Phase I, which took place between April and December 2016, and within which residents of 15 elderly care centres (ECC) in Lisbon were invited to participate. In Phase I, besides undergoing spirometry, fractional exhaled nitric oxide (FeNO) and atopy assessments, participants answered standardised questionnaires and blood samples were collected. In this Phase, every participant also performed a peripheral pulse oximetry.

To be eligible for the OLDER study, participants were required to be ≥65 years of age, present with cognitive and collaboration capabilities sufficient to perform a spirometry and not have any contraindications for lung function testing. The sample size was calculated so as to estimate the frequency of participants with a forced expiratory volume in the 1st second/forced vital capacity (FEV_1_/FVC) of <0.70. According to data published about the Portuguese population, a prevalence of 30% was estimated for this age group. For a confidence level of 95% and a margin of error of 4.5%, it was calculated that 293 participants would have to be studied. According to experience gained in a previous study,^9^ it was considered that about 70% of ECC residents would not meet the inclusion criteria. In order to achieve the target number, plans were made to screen 1000 residents. This paper shall analyse the very elderly patients (≥75 years) included in the study.

The procedures followed were in accordance with the World Medical Association’s Code of Ethics (Declaration of Helsinki). The database was registered with the Portuguese Data Protection Authority. The OLDER study was approved by NOVA Medical School’s Ethics Committee, Portugal (no. 38/2015/CEFCM). The elderly study participants and their caregivers were informed about the study and gave their signed consent.

## Data sources for health assessment

### Questionnaires

In Phase I, participants answered Portuguese versions of standardised questionnaires administered by one single, trained interviewer. These were: (1) The Burden of Obstructive Lung Disease initiative (BOLD) questionnaire,^10,11^ (2) St. George’s Respiratory Questionnaire (SGRQ),^12^ (3) Mini-Mental State Examination (MMSE)^13,14^ and (4) 15-item Geriatric Depression Scale (GDS-15).^15,16^ Authorisations from the authors of these questionnaires were obtained when required.

### Lung function tests

A spirometry with bronchodilatation test was administered and FeNO measurement taken by a trained respiratory therapy technician, as per recommendations.^17,18^ The spirometry was performed using a portable pneumotachograph (Vitalograph^®^ Compact, Vitalograph, Buckingham, UK) and the FeNO using a portable analyser (Niox^®^ Vero, Aerocrine, Solna, Sweden). Because spirometric manoeuvres have been shown to transiently reduce exhaled nitric oxide levels, FeNO analyses were performed before spirometry tests.^18^ Bronchodilation test patients received 400 μg of inhaled salbutamol, administered via a metered-dose inhaler in a spacer. A spirometry test was then repeated after 15 min.^17^ Spirometry results were interpreted alongside the reference equations of the Global Lung Function Initiative 2012.^19^ Spirometry values were only considered valid if all technical standards were achieved and once validated by a committee of experts made up of medical doctors and health technicians.

### Atopy assessment

Atopy was assessed using skin prick reactivity tests to common airborne allergens (Leti^®^, Barcelona, Spain) or, should participants have anergy or refuse the first method, by inhalant panel testing run on blood samples (Phadiatop^®^, Thermo-Fisher Scientific, Uppsala, Sweden).

### Blood biomarkers

A complete blood count (Advia120, Siemens, Munich, Germany) and a high-sensitivity C-reactive protein (hs-CRP) test (Dimension EXL200, Siemens, Munich, Germany) were carried out.

## Variables and applied definitions

The pulmonary treatable traits and definitions included in the study were: airflow limitation (AL; post-bronchodilator z-score FEV_1_/FVC<−1.64),^6,20^ restrictive spirometry pattern (z-score FEV_1_/FVC ≥ +1.64 and z-score FVC<−1.64),^21^ airway smooth muscle contraction (bronchodilator reversibility: an increase of ≥12% and ≥200 mL compared to baseline in either FEV_1_ or FVC)^22^ and type-2 airway inflammation (FeNO >17 ppb – proposed cutoff for elderly patients),^23^ chronic bronchitis (cough with sputum expectoration for at least 3 months a year, for two consecutive years)^20^ and arterial hypoxemia (peripheral oxygen saturation<91%).^24^ Alternatively, cutoff points for type-2 airway inflammation were used: Global Initiative for Asthma (GINA) – FeNO ≥20 ppb^25^; and American Thoracic Society – FeNO >50 ppb.^26^ Taking into consideration that among some patients with normal lung function, including normal FEV_1_, forced mid-expiratory flow (FEF_25%–75%_) may be an early predictive marker of the development of airway obstruction, the frequency of low FEF_25%–75%_ (z-score <−0.8435) was also calculated in patients with normal FEV_1_ (z-score ≥−1.64) pre-bronchodilation.^27^

The extrapulmonary treatable traits considered in this study were: allergic sensitisation (at least one positive skin prick test to common airborne allergens or a positive inhalant panel), anaemia (World Health Organisation definition: haemoglobin levels <13 g/dL for men and <12 g/dL for non-pregnant women^21^), obesity (body mass index ≥30 kg/m^2^),^28^ patients being underweight (body mass index <18.5 kg/m^2^),^28^ cognitive impairments (participants who scored below the validated cutoff points for the Portuguese population in either of the cognitive screening tests - MMSE: 22 for 0–2 years of schooling; 24 for 3–6 years and 27 for 7 years),^14^ depression (GDS-15 > 5)^29^ and chronic systemic inflammation (hs-CRP >3 mg/L).^30^ The behavioural/lifestyle risk factor evaluated was smoking, defined as more than 20-pack-year smoking history.^20^

### Statistical analysis

An exploratory analysis of the variables of interest was carried out for the entire sample. Fisher Exact tests were used to compare differences between categorical variables. A logistic regression was performed to examine the association between extrapulmonary and behavioural/lifestyle treatable traits and an airflow limitation or a restrictive spirometry pattern. The presence of an airflow limitation or restrictive spirometry pattern constituted the outcomes of interest. Age, sex and extrapulmonary and behavioural/lifestyle treatable traits were the exposures considered for the study.

The variables with a *p*-value <0.25 in the bivariate logistic regression analysis were selected for the multivariate logistic regression analysis. The level of significance used was 0.05, although p-values greater than 0.05 and lower than 0.1 were still considered to indicate evidence. Data analysis was performed using STATA (Stata Statistical Software: Release 12; StataCorp LP, Lakeway, TX, USA).

## Results

For this study, 1034 residents were screened with 234 meeting the inclusion criteria and being able to complete all the assessments planned. An inability to participate was mostly due to a lack of cognitive and collaborative capabilities. The median age of participants was 86.3 (IQR: 7.4), and an overwhelming percentage were women (72%; *p* < 0.0001). Among patients assessed, 105 (44.9%; 95% CI: 39.0–51.0) had at least one pulmonary treatable trait, and 17.1% had two or more. The frequency of treatable traits identified is presented in Table 1. Among the patients with pre-bronchodilation normal FEV_1_ (*n* = 121), 76 (62.8%) had a low FEF_25%–75%_.

**Table 1.** Treatable traits in very elderly patients.

	Total (*n* = 234)	Men (*n* = 65)	Women (*n* = 169)	*p*-Value*
Pulmonary treatable traits				
Airflow limitation	10.3	18.5	7.1	**0.02**
Restrictive spirometry pattern	13.2	13.8	13.0	0.83
Airway smooth muscle contraction	13.7	10.8	14.8	0.53
Type-2 airway inflammation	29.5	24.6	31.4	0.34
Chronic bronchitis	15.4	12.3	16.6	0.55
Arterial hypoxemia	1.3	1.5	1.2	1.00
Extra-pulmonary treatable traits				
Allergic sensitisation	20.9	32.8	16.3	**0.01**
Chronic systemic inflammation	47.0	55.4	43.8	0.14
Anaemia	34.4	46.9	29.4	**0.02**
Obesity	27.4	15.4	32.0	0.01
Underweight	1.7	1.5	1.8	1.00
Depression	32.5	26.2	34.9	0.22
Cognitive impairment	20.5	13.8	23.1	0.15
Behavioural/lifestyle risk factors treatable traits				
Smoking	10.7	30.8	3.0	<**0.01**

All the results are expressed in %.

Bold values are statistically significant.

* χ^2^ test.

**Figure 1.**
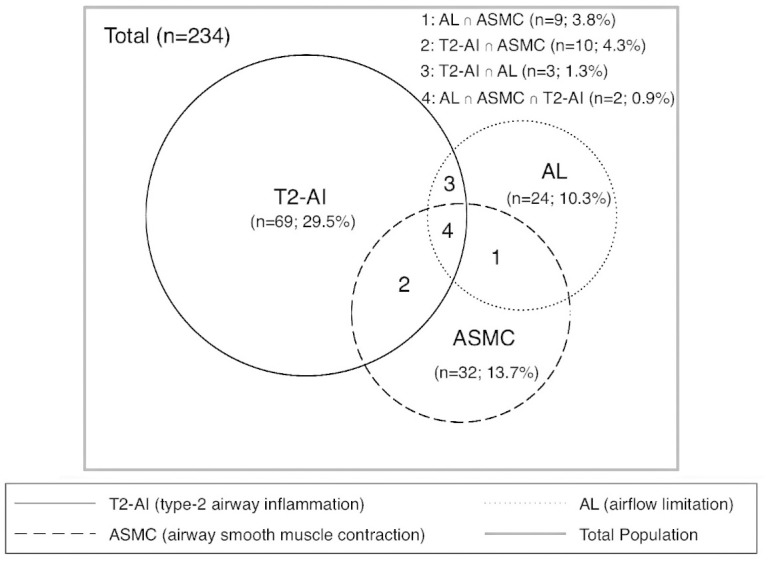
Overlap of participants with airflow limitation, airway smooth muscle contraction and type-2 airway inflammation.

The overlap of participants with airflow limitation, airway smooth muscle contraction and type-2 airway inflammation is shown in Figure 1. When alternative cutoff points for FeNO were used, different frequencies of patients were found: GINA FeNO ≥20 ppb: 25.6%; and American Thoracic Society – FeNO >50 ppb: 2.6%. Of patients with type-2 airway inflammation (*n* = 69; 29.5%), 23.2% had allergic sensitisation and 4.3% had an airflow limitation. Among patients with an airflow limitation (*n* = 24; 10.3%), 37.5% also had airway smooth muscle contraction, 12.5% had concurrent type-2 airway inflammation, and 8.3% had all three coexisting treatable traits.

The frequencies of treatable traits identified in the extrapulmonary and behavioural/risk factor domains are presented in Table 1. Those most frequently identified were: persistent systemic inflammation, anaemia, depression and obesity.

In the sample evaluated, airflow limitation was only significantly linked to smoking of the treatable traits in the extrapulmonary or behavioural/risk factor domains included in the analysis. The associations between airflow limitation and the extrapulmonary and behavioural/risk factor treatable traits identified are presented in Table 2.

**Table 2.** Associations between airflow limitation and the extrapulmonary and behavioural/risk factor treatable traits identified.

	Crude OR (95% CI)	*p*-Value	Adjusted OR* (95% CI)	*p*-Value
Extra-pulmonary treatable traits				
Persistent systemic inflammation	0.95 (0.41–2.21)	0.90	–	–
Allergic sensitisation	2.07 (0.83–5.19)	0.12	2.10 (0.78–5.68)	0.14
Anaemia	1.42 (0.60–3.36)	0.43	–	–
Obesity	0.35 (0.10–1.21)	0.10	0.35 (0.09–1.33)	0.12
Depression	0.51 (0.18–1.44)	0.20	0.69 (0.23–2.03)	0.50
Cognitive impairment	0.76 (0.24–2.32)	0.62	–	–
Behavioural/lifestyle risk factors treatable traits				
Smoking	5.68 (1.70–15.17)	<0.01	5.03 (1.56–16.22)	<0.01

CI: confidence interval; OR: odds-ratio.

* Adjustment for sex (OR: 2.96; *p* = 0.01); age was not significantly associated.

Furthermore, a restrictive spirometry pattern (*n* = 31; 13.2%) was associated with cognitive impairment. The association between a restrictive spirometry pattern and obesity almost fulfilled the statistical significance criteria. Table 3 presents the associations between a restrictive spirometry pattern and the extrapulmonary and behavioural/risk factor treatable traits identified.

**Table 3.** Associations between restrictive spirometry pattern and the extrapulmonary and behavioural/risk factor treatable traits identified.

	Crude OR (95% CI)	*p*-Value	Adjusted OR* (95% CI)	*p*-Value
Extra-pulmonary treatable traits				
Chronic systemic inflammation	1.44 (0.67–3.07)	0.35	–	–
Allergic sensitisation	0.90 (0.35–2.33)	0.82	–	–
Anaemia	1.46 (0.67–3.15)	0.34	–	–
Obesity	2.15 (0.99–4.70)	0.05	2.16 (0.95–4.91)	0.07
Depression	1.37 (0.63–2.99)	0.43	–	–
Cognitive impairment	2.47 (1.09–5.59)	0.03	3.49 (1.42–8.58)	<**0.01**
Behavioural/lifestyle risk factors treatable traits				
Smoking	1.28 (0.41–4.03)	0.67	–	–

CI: confidence interval; OR: odds-ratio.

Bold values are statistically significant.

* Adjustment for age (OR: 0.89; *p* < 0.01); sex was not significantly associated.

## Discussion

In the sample evaluated, a significant number of individuals presented with pulmonary and extrapulmonary treatable traits. A novel association was found between a restrictive spirometry pattern and cognitive impairment. As expected, a large majority of women (72%)^1^ was present in the sample evaluated. On a global level, between 2015 and 2020, life expectancy at birth for women exceeds that of men by 4.8 years in Europe, and by 6.1 years in North America.^1^ Projections indicate that in 2050, women will comprise 54% of the global population aged 65 or over. However, as the sex gap in survival rates between men and women is narrowing, the sex balance among persons aged 80 or over will start to even out.^1^

In the sample evaluated, almost half of the individuals (44.9%) had a pulmonary treatable trait. This fact is in concordance with an ageing global population among which CRDs are becoming a prominent cause of disease.^31^ From the pulmonary treatable traits evaluated, type-2 airway inflammation was the most frequently identified (29.5%), although its unexpectedly high prevalence might be related to the relatively low cutoff applied (17 ppb).^23^ The alternative FeNO cutoff point proposed by GINA^25^ (≥20 ppb) identified a similar number of patients (25.6%) with high FeNO. The percentage of patients identified dropped to 2.6% when the American Thoracic Society’s^26^ proposed cutoff for high FeNO (>50 ppb) was applied. It must be emphasised that the alternative cutoffs proposed for FeNO are tendentiously higher and non-specific to elderly patients when compared with the one chosen.^25^ These results emphasise the need for large population studies to be carried out, in order to strengthen evidence relating to use of this biomarker among this age group. In the sample evaluated, patients with type-2 airway inflammation had a relatively low proportion of allergic sensitisation (23.2%) and airflow limitation (4.3%). If the cutoff used is considered to be trustworthy, it could be speculated that type-2 airway inflammation is frequent in very elderly patients, although rarely associated with allergic mechanisms. Immunosenescence and inflammageing mechanisms relating to the ageing process collectively change innate and adaptive immune responses, with several clinical implications.^32^ Among elderly patients, these implications include decreased vaccination response rates and increased infection rates, as well as altered airways and systemic inflammation.^32^ Higher levels of neutrophils with increased interleukin (IL)-6, IL-8 and C-reactive protein are frequently found among this age group, resembling changes seen in certain severe asthma phenotypes, which are often less responsive to corticosteroid treatment.^32^ In the absence of allergic mechanisms, eosinophilic inflammation can still arise in response to epithelial damage from inhaled pollutants and microbes.^33^ The epithelial alarmins typically released in response to airway epithelial damage promote type-2 airway inflammation independently of allergic mechanisms.^33^ In the sample analysed, a significant proportion of individuals (62.8%) with normal baseline FEV_1_ had low FEF_25-75%_ values. Despite the debate surrounding the clinical relevance of this parameter,^34^ recent evidence postulates that these patients should be monitored carefully, even if they have normal lung function, as they might have an increased incidence rate of COPD in the future.^27^

A high burden of several extrapulmonary treatable traits was found in the sample evaluated, namely persistent systemic inflammation, anaemia, depression and obesity. Anaemia was found to be very frequent (34.4%), and previous reports have also shown that the frequency of anaemia increases with age, especially in elderly men, and exceeds 20% in very elderly patients.^35^ Among this age group, anaemia is typically caused by an underlying aetiology, such as a chronic disease, iron deficiency or myelodysplastic syndromes that can be identified through further investigation.^35^ In this study, the underlying causes of anaemia were not investigated as this was not one of the objectives of the study, but all the information was shared with patients, in order to assist their doctors.

Depression is frequently detected among elderly people, but it is not a normal consequence of ageing.^36^ The prevalence of depression in the sample evaluated (32.5%) is higher than that published for elderly individuals in general.^36^ This fact might be explained by known considerably higher rates of depression among very elderly patients compared to elderly people in general, and among institutionalised older adults.^36^ Assertive approaches to diagnosis and treatment are necessary to improve overall functioning and prevent suicide.^36^ The obesity rate of this sample was similar to what has been published in other studies.^37^ In this study, however, obesity proved to be almost twice as frequent in women than in men (32.0 vs 15.4; *p* = 0.01), contrary to other studies.^37^ Possible explanations for these variations may be related to the relationship between obesity and living place, degree of rurality, socioeconomic status and geography.^37^ Survival-related bias must also be taken into consideration when comparing very elderly patients with elderly patients in general.

In the sample analysed, airflow limitation was only significantly associated with smoking (adjusted odds-ratio 3.82; *p* = 0.02), of the extrapulmonary or behavioural/risk factor treatable traits included in the analysis. Smoking was a treatable trait found in 10.3% of the elderly participants, though it was far more frequent among men (29.2% vs 3.0% in women; *p* < 0.01). Smoking has been defined as one of the greatest public health disasters of the 20th century.^38^ Published reviews have summarised thousands of studies linking smoking to a host of adverse health conditions, including cardiovascular disease, dementia, adverse reproductive outcomes, cancer and respiratory diseases.^38^ Guidelines recommend that clinicians screen individuals who have at least a 20-pack-year smoking history, as defined in this study as a treatable trait.^20^ Smoking can affect airways by inducing tissue damage both directly, through oxidative stress, and indirectly, by eliciting an inflammatory response.^39^ The severity of airflow limitation is associated with the extent to which lung tissue damage has occurred.^39^ Airway remodelling thickens airway walls in a manner that involves the epithelium, lamina propria, smooth muscle and adventitia of airway walls that have a diameter of less than 2 mm.^39^ Regarding the epidemiology of smoking, as a treatable trait, it is a well-known fact that far more men than women use tobacco products.^39^ Participants of this study were mostly born in the early 20th century, when smoking was a habit observed far more frequently among men. As such, this result met expectations. However, the epidemic of tobacco use among women is increasing, which means this sex gap will likely diminish.^40^ The epidemic of smoking among women should draw the attention of all in the future.

A restrictive spirometry pattern was found in 13.2% of the participants in this study. This treatable trait frequency was comparable to that found in previous studies carried out with similar populations and evaluative methods.^41^ The restrictive spirometry pattern was significantly associated with cognitive impairment. In this study, obesity was not linked to a restrictive spirometry pattern (adjusted odds-ratio: 2.16; *p* = 0.07), although the respiratory consequences of being overweight are well established.^42^ Patients present with predominantly mechanical consequences, though an inflammatory component is also present.^42^ Adiposity on the thoracic cage and abdomen can affect chest wall movement, airway size, respiratory muscle function and lung perfusion.^42^ From a public health perspective, it is fundamental to encourage lifestyle changes in elderly patients. Physicians will need to balance the potential danger of weight loss in older persons against the complications of obesity in order to decide on the best patient-centred approach. One clear recommendation is that all weight loss regimens in the elderly need to be coupled with a comprehensive resistance exercise program.

The results of this study excitingly support recent evidence about the link between restrictive pattern and cognitive impairment (adjusted odds-ratio: 3.89; *p* < 0.01). As previously published by Lutsey et al., ^43^ restrictive spirometry pattern has been linked to cognitive impairment independently of smoking status and was more pronounced than in patients with airflow limitation. The evidence available supports the hypothesis that there is a link between restrictive spirometry pattern and cognitive impairment, both for Alzheimer’s disease and cerebrovascular aetiologies, although this was not evaluated in this study.^43^ Restrictive impairment patients tend to have ventilation-perfusion mismatch and hypoxemia, although further research is needed in order to gain an understanding of the full mechanisms involved.^43^

This study has some limitations that may be prejudicial to conclusions. Although the OLDER study was a one-year prospective cohort study, the analysis included in this paper was only cross-sectional, using patients’ baseline data. Additionally, the sample of participants was not large enough to find a considerable number of patients with pulmonary treatable traits. Furthermore, we do not know to what extent these results could be extrapolated to the general population, as the participants were recruited in ECC and a large number of participants were considered ineligible. The major strengths of our study are the inclusion of a carefully selected sample of very old persons recruited in a non-clinical setting, a detailed clinical characterisation of terms and the precise definition of treatable traits in very elderly patients. To the best of the authors’ knowledge, this is the first study providing such information, finding associations between pulmonary, extrapulmonary and behavioural/lifestyle risk factors in this age group.

## Conclusions

Very elderly patients have a high burden of several differing treatable traits. Almost half of the individuals tested had a pulmonary treatable trait, a prominent issue among an ageing population. This study contributes exciting new data about the association between a restrictive spirometry pattern and cognitive impairment. Clinical research about elderly patients is fundamental to providing proper care to a particularly vulnerable age group.

## Supplemental Material

sj-pdf-1-sci-10.1177_00368504211013171 – Supplemental material for Ventilatory defects and treatable traits in very elderly patients Click here for additional data file. Supplemental material, sj-pdf-1-sci-10.1177_00368504211013171 for Ventilatory defects and treatable traits in very elderly patients by João Gaspar-Marques, Teresa Palmeiro, Iolanda Caires, Paula Leiria Pinto, Nuno Neuparth and Pedro Carreiro-Martins in Science Progress

sj-xlsx-2-sci-10.1177_00368504211013171 – Supplemental material for Ventilatory defects and treatable traits in very elderly patients Click here for additional data file. Supplemental material, sj-xlsx-2-sci-10.1177_00368504211013171 for Ventilatory defects and treatable traits in very elderly patients by João Gaspar-Marques, Teresa Palmeiro, Iolanda Caires, Paula Leiria Pinto, Nuno Neuparth and Pedro Carreiro-Martins in Science Progress
