# Supplementary material for: Ventilatory defects and treatable traits in very elderly patients
Source: Sci Prog. 2021 Apr 30;104(2):00368504211013171. doi: 10.1177/00368504211013171 (PMC10305819; doi:10.1177/00368504211013171)
Supplement: sj-pdf-1-sci-10.1177_00368504211013171 – Supplemental material for Ventilatory defects and treatable traits in very elderly patients [file sj-pdf-1-sci-10.1177_00368504211013171.pdf]

A Preencher pelo Inquiridor

Data: [ ][ ] - [ ][ ] - [ ][ ][ ][ ]

Hora de Início: [ ][ ] : [ ][ ]

ID do Inquiridor: [ ][ ]

SOCIO-DEMOGRÁFICO

1. **Sexo**: ○ Feminino ○ Masculino

2. Em que **ano nasceu**? [ ][ ][ ][ ]

3. Qual é o seu **estado civil**? ○ Solteiro ○ Casado/União de Facto ○ Separado/Divorciado ○ Viúvo

4. Que **estudos completou**? [ ][ ] **anos completos**

○ Analfabeto ○ 1º a 4º anos ○ 7º a 9º anos ○ Estudos univers./form. pós-grad.

○ Sabe ler e/ou escrever ○ 5º a 6º anos ○ 10º a 12º anos

5. Qual a sua **profissão**? (Principal) [ ]

6. **Onde nasceu**? (Distrito) [ ]

7. **Migrou**: ○ Não ○ Sim ○ Não Sabe

Se migrou, especifique? (Distrito, País) [ ]

8. **Duração da residência atual** [ ][ ] **anos**

SAÚDE:

9. **Contenção Física Observável**: ○ Não ○ Sim

Vamos falar agora da sua saúde

	Muito Má	Má	Nem boa nem má	Boa	Muito Boa
10a. Como **avalia** a sua **saúde**?	○	○	○	○	○

	Muito insatisfeito	Insatisfeito	Nem satisfeito nem insatisfeito	Satisfeito	Muito satisfeito
10b. Até que ponto está **satisfeito(a)** com a sua **saúde**?	○	○	○	○	○

	Negativo	Positivo
10c. Se **saúde** "Nem boa nem má", "Nem satisfeito nem insatisfeito", **considera isso negativo ou positivo**?	○	○

QUEIXAS RESPIRATÓRIAS (BOLD)

Estas questões são essencialmente sobre o seu peito. Por favor assinale se possível, "Sim" ou "Não". Em caso de dúvida sobre a resposta, por favor assinale "N/S".

TOSSE

12. Costuma ter **tosse**, habitualmente? ○*Não* ○*Sim* ○*N/S*
*[Se **NÃO** ou **NÃO SABE**, passe para a **Questão 16**.]*

13. Costuma tossir **4 a 6 vezes ao dia, 4 ou mais dias da semana**? ○*Não* ○*Sim* ○*N/S*

14. Costuma tossir dessa forma na **maioria dos dias**, durante **3 ou mais meses do ano**? ○*Não* ○*Sim* ○*N/S*

15. Desde **há quantos anos** tem esta tosse? [|] anos ○<2 ○2 a 5 ○> 5

EXPECTORAÇÃO (CATARRO)

16. Costuma deitar fora **expectoração** (catarro) do seu peito? ○*Não* ○*Sim* ○*N/S*
Considere a expectoração associada ao primeiro cigarro ou quando sai para a rua.
Exclua as secreções provenientes do nariz. Valorize a que é engolida
[Se respondeu **NÃO** ou **NÃO SABE**, passe para a **Questão 20**.]

17. Costuma expectorar dessa maneira **pelo menos duas vezes ao dia, 4 ou mais dias da semana**? ○*Não* ○*Sim* ○*N/S*

18. Costuma expectorar dessa forma, na **maioria dos dias,** durante **3 ou mais meses consecutivos do ano**? ○*Não* ○*Sim* ○*N/S*

19. **Há quantos anos** tem essa expectoração? [|] anos ○<2 ○2 a 5 ○> 5

PIEIRA (SILVOS NO PEITO)

20. Já alguma vez teve um ataque de **pieira** (silvos no peito) que o tenha feito sentir dificuldade em respirar? ○*Não* ○*Sim* ○*N/S*
[Se respondeu **NÃO** ou **NÃO SABE,** passe para a **Questão 25.**]

21. Que **idade** tinha quando teve o **primeiro ataque de pieira** no peito? ☐☐ anos

22. Já teve **dois ou mais episódios**? ○*Não* ○*Sim* ○*N/S*

23. Já alguma vez **necessitou de medicamentos ou tratamento** para esse(s) ataque(s)? ○*Não* ○*Sim* ○*N/S*

24. Teve alguma vez **pieira nos últimos 12 meses**? ○*Não* ○*Sim* ○*N/S*

DIFICULDADE EM RESPIRAR

25. Tem limitações do andar, por dificuldade em respirar, por outra situação não relacionada com o coração e os pulmões? ○*Não* ○*Sim* ○*N/S*

26. Se **SIM**, especifique e ***passe para a Questão 32.*** ☐

FALTA DE AR

27. Costuma sentir-se atrapalhado pela **falta de ar** quando **anda mais depressa** ou quando **sobe uma pequena inclinação**? ○*Não* ○*Sim* ○*N/S*
[Se respondeu **NÃO** ou **NÃO SABE**, passe para a **Questão 32.**]

28. Tem que **andar mais devagar** do que as pessoas da sua idade em **terreno plano**, devido à falta de ar? ○*Não* ○*Sim* ○*N/S*

29. Já alguma vez teve que **parar para respirar** quando caminha no seu próprio passo, em terreno plano? ○*Não* ○*Sim* ○*N/S*

30. Já alguma vez teve que **parar para respirar após andar cerca de 100 metros** (ou após andar alguns minutos) em terreno plano? ○*Não* ○*Sim* ○*N/S*

31. Sente-se **impedido de sair à rua** pela falta de ar ou sente falta de ar enquanto se **veste ou despe**? ○*Não* ○*Sim* ○*N/S*

DOENÇAS RESPIRATÓRIAS (BOLD)

ENFISEMA

32. Já algum médico lhe disse que tinha **enfisema**? ○*Não* ○*Sim* ○*N/S*

ASMA

33. Já algum médico lhe disse que tinha **asma**? ○*Não* ○*Sim* ○*N/S*
    [Se respondeu NÃO ou **NÃO SABE*, passe para a Questão 37.]***
34. **Ainda tem** asma? ○*Não* ○*Sim* ○*N/S*
35. Com que **idade começou** a asma? ☐☐ anos
36. **Se já não tem** asma, com que **idade deixou de ter queixas**? ☐☐ anos

TUBERCULOSE

37. Já algum médico lhe disse que teve **tuberculose**? ○*Não* ○*Sim* ○*N/S*
    [Se respondeu NÃO ou **NÃO SABE*, passe para a Questão 41.]***
38. **Ainda tem** tuberculose? ○*Não* ○*Sim* ○*N/S*
39. Com que **idade começou** a tuberculose? ☐☐ anos
40. **Se já não tem** tuberculose, com que **idade deixou de ter queixas**? ☐☐ anos

PNEUMONIA

41. Já algum médico lhe disse que tinha **pneumonia**? ○*Não* ○*Sim* ○*N/S*
    [Se respondeu NÃO ou **NÃO SABE*, passe para a Questão 45.]***
42. **Ainda tem** pneumonia? ○*Não* ○*Sim* ○*N/S*
43. Com que **idade começou** a pneumonia? ☐☐ anos
44. **Se já não tem** pneumonia, com que **idade deixou de ter queixas**? ☐☐ anos

RINITE ALÉRGICA

45. Já algum médico lhe disse que tinha **rinite alérgica**? ○*Não* ○*Sim* ○*N/S*
    [Se respondeu NÃO ou **NÃO SABE*, passe para a Questão 49.]***
46. **Ainda tem** rinite alérgica? ○*Não* ○*Sim* ○*N/S*
47. Com que **idade começou** a rinite alérgica? ☐☐ anos
48. **Se já não tem** rinite alérgica, com que **idade deixou de ter queixas**? ☐☐ anos

OUTRAS DOENÇAS RESPIRATÓRIAS

49. Já algum médico lhe disse que tinha **outra doença respiratória**? ○*Não* ○*Sim* ○*N/S*
    ***[Se respondeu NÃO** ou **NÃO SABE**, passe para a Questão 54.]*

50. Se **SIM**, especifique

51. **Ainda tem** essa doença respiratória? ○Não ○Sim

52. Com que **idade começou** essa doença respiratória? |__|__| anos

53. **Se já não tem** essa doença respiratória, com que **idade deixou de ter queixas**? |__|__| anos

PROBLEMAS CARDÍACOS

54. Já algum médico lhe disse que tinha **problemas do coração**? ○*Não* ○*Sim* ○*N/S*

55. Fez algum **tratamento para problemas cardíacos** nos últimos 10 anos? ○*Não* ○*Sim* ○*N/S*

56. Já algum médico lhe disse que teve um **ataque cardíaco** (enfarte do miocárdio, obstrução coronária, trombose coronária)? ○*Não* ○*Sim* ○*N/S*

AMBIENTE E TABAGISMO

57. Alguma vez trabalhou durante um ano ou mais num **lugar poeirento**? ○*Não* ○*Sim* ○*N/S*

58. **Quantos anos** trabalhou nesse emprego? |__|__| anos

59. Já alguma vez **fumou cigarros**? ***[Se respondeu NÃO, passe para a Questão 65.]*** ○Não ○Sim
    (**Não** significa menos de 20 maços de cigarro ou 340g de tabaco durante a sua vida ou menos de 1 cigarro por dia durante um ano).

60. **Fuma cigarros** (atualmente/no último mês)? ○Não ○Sim

61. Com que **idade começou** a fumar habitualmente? |__|__| anos

62. **Se parou** de fumar cigarros por completo, que **idade tinha quando parou**? |__|__| anos

63. Quantos **cigarros fuma por dia** atualmente? |__|__| cig/d

64. Em média, durante todo o tempo em que fumou, **quantos cigarros fumou por dia**? |__|__| cig/d

SAÚDE RESPIRATÓRIA – 3 meses

As próximas perguntas, referem-se aos últimos 3 meses. Por favor responda se possível, "Sim" ou "Não". Em caso de dúvida sobre a resposta, por favor responda "N/S".

*Q1. Nos últimos 3 meses, esteve alguma vez **doente com tosse**?*<br>*[Se **NÃO** ou **NÃO SABE**, passe para a **Q3**.]*	○*Não* ○*Sim* ○*N/S*
*Q2. Nos últimos 3 meses, necessitou de **consultar um médico devido à tosse**?*	○*Não* ○*Sim* ○*N/S*
*Q3. Nos últimos 3 meses, esteve alguma vez **doente com pieira (silvos no peito)**?*<br>*[Se **NÃO** ou **NÃO SABE**, passe para a **Q5**.]*	○*Não* ○*Sim* ○*N/S*
*Q4. Nos últimos 3 meses, necessitou de **consultar um médico devido à pieira?***	○*Não* ○*Sim* ○*N/S*
*Q5. Nos últimos 3 meses, esteve alguma vez **constipado**?*<br>*[Se **NÃO** ou **NÃO SABE**, passe para a **Q7**.]*	○*Não* ○*Sim* ○*N/S*
*Q6. Nos últimos 3 meses, necessitou de **consultar um médico devido à constipação?***	○*Não* ○*Sim* ○*N/S*
*Q7. Nos últimos 3 meses, esteve alguma vez **com uma infeção respiratória,** dos brônquios ou pulmões?*<br>*[Se **NÃO** ou **NÃO SABE**, passe para a **Q9**.]*	○*Não* ○*Sim* ○*N/S*
*Q8. Nos últimos 3 meses, necessitou de **consultar um médico devido à infeção respiratória,** dos brônquios ou pulmões?*	○*Não* ○*Sim* ○*N/S*
*Q9. Nos últimos 3 meses, necessitou de **tomar antibiótico** por algum problema dos brônquios ou pulmões?*	○*Não* ○*Sim* ○*N/S*
Q10. Nos últimos 3 meses, esteve alguma vez ***internado***?	○*Não* ○*Sim* ○*N/S*
Q11. Se **SIM**, especifique	

A Preencher pelo Cardiopneumologista

Q12. Avaliação Diagnóstica Complementar

Q12a. Pressão arterial _____ /_____mmHg

Q12b. Faz medicação para a HTA? ○ Não ○ Sim ○ Não Sabe

Q12c. SaO_2 _____ %

Q12d. FC _____ bpm

Q12e. Altura _____ cm | Envergadura _____ cm (Altura ♂=Envergadura/1.06 | Altura ♀=Envergadura/1.03)

Q12f. Peso _____ Kg

Q12g. Espirometria ○ Sim Colaboração:____

○ Não Motivo: ____

COGNIÇÃO (MMS)
Vou fazer-lhe agora outras perguntas.

Orientação Temporal

65. Em que ano estamos?	○ Certo ○ Errado		○ N/S	○ N/R
66. Em que mês estamos?	○ Certo ○ Errado		○ N/S	○ N/R
67. Quantos são hoje (dia do mês)?	○ Certo ○ Errado		○ N/S	○ N/R
68. Em que estação do ano estamos?	○ Certo ○ Errado		○ N/S	○ N/R
69. Que dia da semana é hoje?	○ Certo ○ Errado		○ N/S	○ N/R

Orientação Espacial

70. Como se chama o nosso país?	○ Certo ○ Errado		○ N/S	○ N/R
71. Em que distrito vive?	○ Certo ○ Errado		○ N/S	○ N/R
72. Em que terra vive?	○ Certo ○ Errado		○ N/S	○ N/R
73. Como se chama esta casa?	○ Certo ○ Errado		○ N/S	○ N/R
74. Em que andar estamos?	○ Certo ○ Errado		○ N/S	○ N/R

Retenção

Agora vou dizer-lhe 3 palavras. Queria que as repetisse e que procurasse decorá-las, porque dentro de alguns minutos vou pedir-lhe que me diga essas 3 palavras. As palavras são: Pêra, Gato, Bola. Repita as 3 palavras.
Repetir todas as palavras até serem totalmente aprendidas, num máximo de 6 tentativas. Se as palavras não forem aprendidas não se pode fazer a Evocação.

75. Pêra	○ Certo ○ Errado		○ N/S	○ N/R
76. Gato	○ Certo ○ Errado		○ N/S	○ N/R
77. Bola	○ Certo ○ Errado		○ N/S	○ N/R

Atenção e Cálculo

Agora peço-lhe que me diga quantos são 30 menos 3 e que ao número encontrado volte a subtrair 3, até eu lhe dizer para parar.
Parar ao fim de 5 respostas. Se fizer um erro na subtração, mas continuar a subtrair corretamente a partir do erro, conta-se como um único erro.

78. 1º cálculo	○ Certo ○ Errado		○ N/S	○ N/R
79. 2º cálculo	○ Certo ○ Errado		○ N/S	○ N/R
80. 3º cálculo	○ Certo ○ Errado		○ N/S	○ N/R
81. 4º cálculo	○ Certo ○ Errado		○ N/S	○ N/R
82. 5º cálculo	○ Certo ○ Errado		○ N/S	○ N/R

Se o sujeito não realizar ou errar em mais do que 3 questões do cálculo anterior, realizar este teste.

Vou dizer-lhe 3 números e queria que me dissesse esses 3 números, mas ao contrário, isto é do último para o primeiro. Os números são: 5 9 2

83. 1º resposta ○ Certo ○ Errado [ ] ○ N/S ○ N/R

84. 2º resposta ○ Certo ○ Errado [ ] ○ N/S ○ N/R

85. 3º resposta ○ Certo ○ Errado [ ] ○ N/S ○ N/R

Evocação

Agora veja se me consegue dizer quais foram as 3 palavras que lhe pedi há pouco para decorar.

Só se efetua se o sujeito tiver aprendido as 3 palavras da prova de retenção.

86. Pêra ○ Certo ○ Errado [ ] ○ N/S ○ N/R

87. Gato ○ Certo ○ Errado [ ] ○ N/S ○ N/R

88. Bola ○ Certo ○ Errado [ ] ○ N/S ○ N/R

Nomeação

Como se chama isto?

89. Relógio ○ Certo ○ Errado [ ] ○ N/S ○ N/R

90. Lápis ○ Certo ○ Errado [ ] ○ N/S ○ N/R

Repetição da frase

91. Repita a frase: "**O rato rói a rolha**" ○ Certo ○ Errado [ ] ○ N/S ○ N/R

Compreensão verbal

Vou dar-lhe uma folha de papel. Quando eu lhe entregar o papel (**entregar com ambas as mãos**):
pegue nele com a sua mão direita, dobre-o ao meio e coloque-o sobre a mesa.

92. Mão direita ○ Certo ○ Errado [ ] ○ N/S ○ N/A

93. Dobrar ao meio ○ Certo ○ Errado [ ] ○ N/S ○ N/A

94. Colocar sobre a mesa ○ Certo ○ Errado [ ] ○ N/S ○ N/A

Compreensão de leitura

Mostrar a frase num cartão. Se o sujeito for analfabeto, o inquiridor deverá ler-lhe a frase.

95. Leia e cumpra o que diz neste cartão ○ Certo ○ Errado [ ] ○ N/S ○ N/A

Escrever frase

Deverá ter sujeito, verbo e ter sentido. Erros gramaticais e troca de letras não contam.

96. Escreva uma frase ○ Certo ○ Errado [ ] ○ N/S ○ N/A

Fazer desenho

Mostrar o desenho num cartão. Devem estar presentes os 10 ângulos e 2 deles devem estar intersetados. Tremor e erros de rotação não contam.

97. Copie o desenho que lhe vou mostrar. ○ Certo ○ Errado [ ] ○ N/S ○ N/A

96. Escrever frase

97. Fazer desenho

Este questionário procura conhecer a sua qualidade de vida, saúde, e outras áreas da sua vida. Peço-lhe para responder a todas as perguntas. Se não tiver a certeza da resposta a dar a uma pergunta, escolha a que lhe parecer mais apropriada. Esta pode muitas vezes ser a resposta que lhe vier primeiro à cabeça. Pedimos-lhe que tenha em conta a sua vida nas 2 últimas semanas.

QUALIDADE DE VIDA (WHOQoL-BREF):

	Muito Má	Má	Nem boa nem má	Boa	Muito Boa
98. Como **avalia** a sua **qualidade de vida**?	○	○	○	○	○

	Negativo	Positivo
99. Se **QV** “Nem boa nem má” **considera isso negativo ou positivo**?	○	○

As perguntas que se seguem são para ver até que ponto **sentiu certas coisas** nas 2 últimas semanas

	Nada	Pouco	Nem muito nem pouco	Muito	Muitíssimo
100. Em que medida **as suas dores (físicas) o(a) impedem de fazer o que precisa de fazer**?	○	○	○	○	○
101. Em que medida **precisa de cuidados médicos para fazer a sua vida diária**?	○	○	○	○	○
102. Até que ponto **gosta da sua vida**?	○	○	○	○	○
103. Em que medida **sente que a sua vida tem sentido**?	○	○	○	○	○
104. Até que ponto **se consegue concentrar**?	○	○	○	○	○
105. Em que medida **se sente em segurança no seu dia a dia**?	○	○	○	○	○
106. Em que medida **é saudável o seu ambiente físico**?	○	○	○	○	○

As perguntas que se seguem são para ver até que ponto **experimentou ou foi capaz de fazer certas coisas** nas 2 últimas semanas

	Nada	Pouco	Moderadamente	Bastante	Completamente
107. Tem **energia** suficiente **para a sua vida diária**?	○	○	○	○	○
108. É capaz de **aceitar** a sua **aparência física**?	○	○	○	○	○
109. Tem **dinheiro suficiente para** satisfazer as suas **necessidades**?	○	○	○	○	○
110. Até que ponto tem **fácil acesso às informações necessárias para** organizar a **sua vida diária**?	○	○	○	○	○
111. Em que medida **tem oportunidade para** realizar **atividades de lazer** (se entreter)?	○	○	○	○	○

	Muito Má	Má	Nem boa nem má	Boa	Muito Boa
112. Como **avaliaria** a sua **mobilidade** (capacidade para se movimentar e deslocar por si próprio**)**?	○	○	○	○	○

<u>As perguntas que se seguem</u> destinam-se a avaliar se **se sentiu bem ou satisfeito(a) em relação a vários aspectos da sua vida** nas 2 últimas semanas

	Muito insatisfeito	Insatisfeito	Nem satisfeito nem insatisfeito	Satisfeito	Muito satisfeito
113. Até que ponto está satisfeito(a) com o **seu sono**?	○	○	○	○	○
114. Até que ponto está satisfeito(a) **com a sua capacidade para desempenhar as atividades do seu dia-a-dia**?	○	○	○	○	○
115. Até que ponto está satisfeito(a) **com a sua capacidade de trabalho**?	○	○	○	○	○
116. Até que ponto está satisfeito(a) **consigo próprio**?	○	○	○	○	○
117. Até que ponto está satisfeito(a) **com as suas relações pessoais**?	○	○	○	○	○
118. Até que ponto está satisfeito(a) **com a sua vida sexual**?	○	○	○	○	○
119. Até que ponto está satisfeito(a) **com o apoio que recebe dos seus amigos**?	○	○	○	○	○
120. Até que ponto está satisfeito(a) **com as condições do lugar em que vive**?	○	○	○	○	○
121. Até que ponto está satisfeito(a) **com o acesso que tem aos serviços de saúde**?	○	○	○	○	○
122. Até que ponto está satisfeito(a) **com os transportes que utiliza**?	○	○	○	○	○

Estamos quase a finalizar este questionário, mas antes de terminar gostaríamos de saber como se sente (ao nível das suas emoções) no seu dia-a-dia.

	Nunca	Poucas vezes	Algumas vezes	Frequentemente	Sempre
123. Com que frequência tem **sentimentos negativos**, tais como **tristeza**, **desespero**, **ansiedade** ou **depressão**?	○	○	○	○	○

<u>DEPRESSÃO</u> (GDS15)

124. Está **satisfeito(a) com a sua vida**?	○ Não	○ Sim
125. Pôs de lado muitas das suas **atividades e interesses**?	○ Não	○ Sim
126. Sente a sua **vida vazia**?	○ Não	○ Sim
127. Fica muitas vezes **aborrecido(a)**?	○ Não	○ Sim
128. **Está bem disposto(a)** a maior parte do tempo?	○ Não	○ Sim
129. **Tem medo** que lhe vá acontecer alguma coisa de mal?	○ Não	○ Sim
130. **Sente-se feliz** a maior parte do tempo?	○ Não	○ Sim
131. **Sente-se** muitas vezes **desamparado(a)**?	○ Não	○ Sim
132. **Prefere ficar** no Lar, **em vez de sair e fazer coisas novas**?	○ Não	○ Sim
133. Acha que tem mais **problemas de memória** do que as outras pessoas?	○ Não	○ Sim
134. Pensa que **é bom estar vivo(a)**?	○ Não	○ Sim
135. Sente-se **inútil**?	○ Não	○ Sim
136. Sente-se **cheio(a) de energia**?	○ Não	○ Sim
137. Sente que para si **não há esperança**?	○ Não	○ Sim
138. Pensa que a situação da maioria **das pessoas é melhor do que a sua**?	○ Não	○ Sim

139. Este questionário foi preenchido

- ○ Respondeu autonomamente
- ○ Com ajuda do cuidador
- ○ Com ajuda de familiar
- ○ Com ajuda de cuidador e familiar

Hora de fim: ☐☐ : ☐☐

Informação adicional - opcional

